# Relapsing COVID-19 in a Patient With Non-Hodgkin Lymphoma on Chemotherapy

**DOI:** 10.7759/cureus.49974

**Published:** 2023-12-05

**Authors:** António Carujo, Luís Ferreira, Rui Bergantim, André Santos Silva, António Ludgero Vasconcelos

**Affiliations:** 1 Infectious Diseases Department, Santo António University Hospital, Porto, PRT; 2 Clinical Hematology Department, São João University Hospital, Porto, PRT; 3 Faculty of Medicine, University of Porto, Porto, PRT; 4 Hemato-Oncology Department, Lusíadas Hospital of Porto, Porto, PRT; 5 Abel Salazar Biomedical Sciences Institute, University of Porto, Porto, PRT

**Keywords:** rituximab, lymphoma, r-chop protocol, immunosuppression therapy, covid-19

## Abstract

Hematologic malignancies and chemotherapy are risk factors for COVID-19 progression and mortality. Immunocompromised hosts, particularly those with severe B-cell depletion, can shed viable viruses for extended periods, which can lead to persistent infection. We present the case of a 73-year-old male with diffuse large B-cell lymphoma (stage IV-B) under curative immunochemotherapy with rituximab, cyclophosphamide, doxorubicin, vincristine, and prednisone (R-CHOP). After the first episode of mild COVID-19, he developed two severe relapses following the third and fourth cycles of R-CHOP. Lung CT scans performed in both episodes showed new-onset ground-glass infiltrates and fibrosis of previously affected pulmonary segments. In light of similar semiquantitative SARS-CoV-2 viral loads between episodes, without further risk exposure or microbiological findings, persistent COVID-19 with severe clinical relapses was assumed and successfully treated with polyclonal immunoglobulin and remdesivir. Whole-genome sequencing was performed in all samples, confirming the same specimen, which belonged to the B.1.177 lineage. This case stands out for the unusually long viral persistence and the various relapses of severe COVID-19 related to the worsening immune status with each immunochemotherapy cycle.

## Introduction

COVID-19 in immunocompromised hosts differs in disease severity and shedding duration [1,2]. Active cancer is a risk factor for progression to severe COVID-19 and mortality, particularly with hematologic malignancies and their myelosuppressive immunochemotherapy regimens [1,3]. Effective vaccination and better therapies have improved overall outcomes [4,5].

Immunocompromised hosts can shed viable viruses for prolonged periods of time, regardless of disease severity [2]. This can lead to persistent active or relapsing infection, particularly in those with severe B-cell depletion due to cancer therapy with rituximab [6,7]. Important public health and infection control implications arise, with the duration of infection prevention precautions varying between protocols.

## Case presentation

A 73-year-old male with diffuse large B-cell lymphoma (stage IV-B) was started on curative immunochemotherapy with rituximab, cyclophosphamide, doxorubicin, vincristine, and prednisone (R-CHOP) in January 2021. Later that month, he developed anosmia and was diagnosed with mild COVID-19, in the presence of a normal chest x-ray. Supportive treatment was given, as oral antivirals and monoclonal antibodies were not yet available.

**Figure 1:**
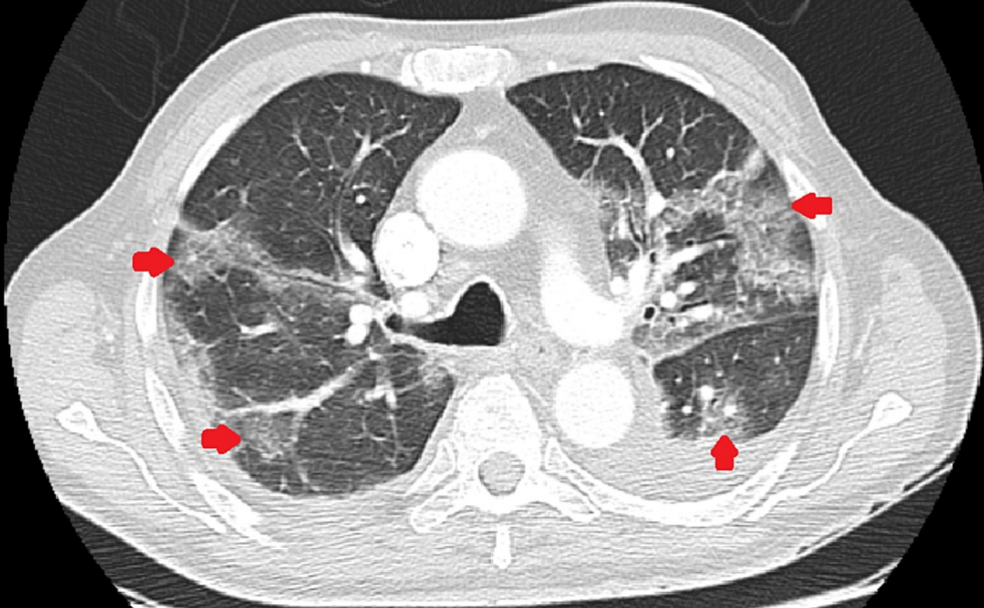
Thoracic CT angiography performed in March 2021 Red arrows: bilateral multifocal ground-glass opacities, with adjacent interstitial densification, presenting a crazy paving pattern.

In March, after the third cycle of R-CHOP, he developed fever, non-productive cough, and minimal exertion dyspnea, presenting with hypoxemic respiratory failure (pO_2_ 58 mmHg and pCO_2_ 41 mmHg, FiO_2_ 21%), pancytopenia with neutropenia (leukopenia 650/µL), and a C-reactive protein of 200 mg/L. A thoracic CT angiography revealed bilateral multifocal ground-glass opacities with adjacent interstitial densification; there was no pulmonary thromboembolism (Figure 1). The patient was hospitalized, and given the suggestive CT scan, severe COVID-19 was considered and dexamethasone was started. Other differential diagnoses included bacterial pneumonia, with risk factors for nosocomial infection given the frequent hospital visits for chemotherapy, and pneumocystosis due to his combined immunosuppression with corticosteroids, so the patient was further treated with imipenem and cotrimoxazole. Rarer diagnosis considered included rituximab-associated pneumonitis and doxorubicin-associated heart failure, excluded by low pro-brain natriuretic peptide values. Blood and urine cultures were sterile and pneumococcal and *Legionella* urinary antigen tests were negative. There were no microbiological findings other than a persistent positive PCR for SARS-CoV-2. The patient evolved favorably with no need for mechanical ventilation, presenting resolution of the respiratory failure and leukocyte recovery.

**Figure 2:**
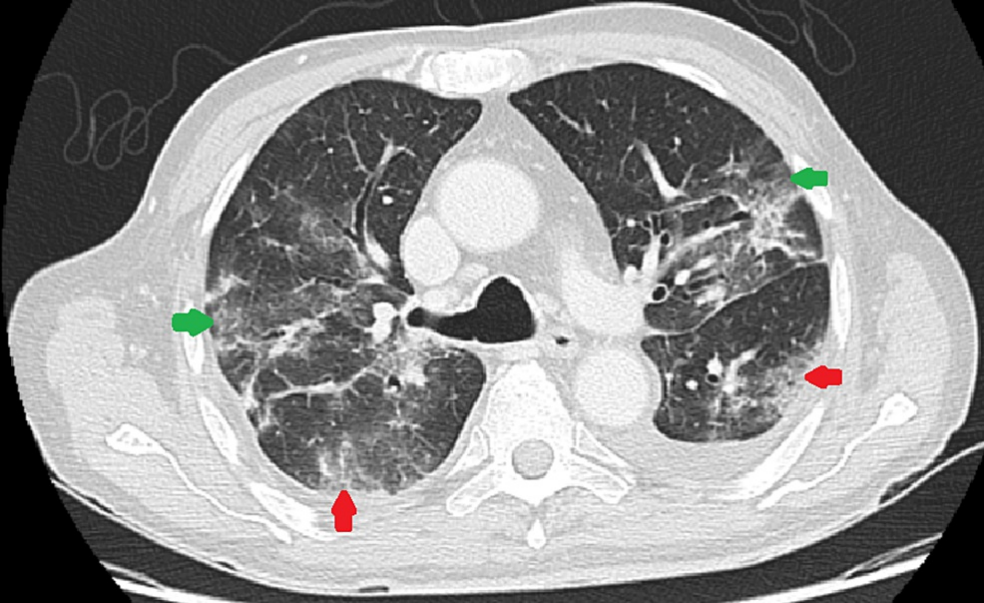
Thoracic CT scan performed in April 2021 Green arrows: fibrosis and bronchiectatic evolution of the previously affected pulmonary segments; red arrows: new ground-glass infiltrates in previously spared areas.

After the fourth cycle of R-CHOP in April, the exact same symptoms resumed and he again presented respiratory failure (pO_2_ 53 mmHg and pCO_2_ 43 mmHg, FiO_2_ 21%). A thoracic CT scan was repeated, which was vital for the clinical analysis of the patient, showing fibrosis and bronchiectatic evolution of previously affected pulmonary segments, with new ground-glass infiltrates in previously spared areas (Figure 2). Further workup revealed decreased B-lymphocyte counts and agammaglobulinemia. Once again, the only microbiological finding was a positive PCR for SARS-CoV-2. In light of a similar semiquantitative analysis of SARS-CoV-2 viral load between episodes (cycle thresholds of 20), without new risk exposure, in a patient with a persistent state of immunosuppression aggravated by each chemotherapy cycle, we assumed a persistent COVID-19 infection with clinical relapse. We opted for polyclonal immunoglobulin supplementation and remdesivir cycle. The patient evolved favorably with no need for mechanical ventilation, presenting resolution of the respiratory failure, and was discharged. Considering the apparent cancer remission on the PET scan, we opted for chemotherapy suspension until viral eradication and radiological resolution of the pulmonary activity, to prevent further readmissions.

**Figure 3:**
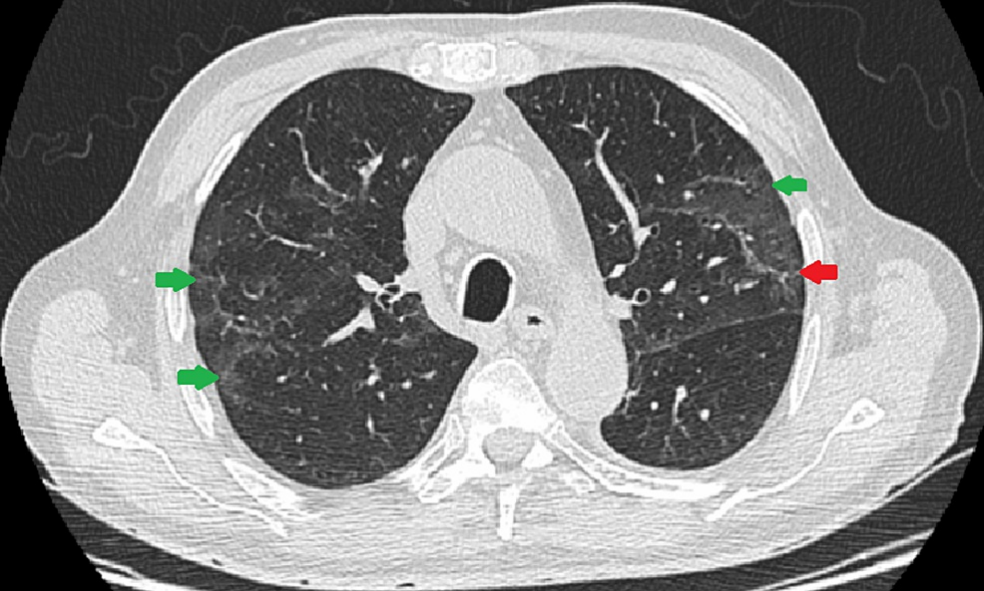
Thoracic CT scan performed in June 2021 Green arrows: fibrosis of the previously affected segments; red arrow: maintenance of some of the ground-glass infiltrates.

PCR for SARS-CoV-2 was subsequently performed monthly. A thoracic CT scan was repeated in June, revealing fibrosis of most previously affected segments, but also the persistence of some ground-glass infiltrates (Figure 3). The patient was consequently submitted to another cycle of immunoglobulin supplementation. In August, seven months later, the patient finally presented a negative PCR result. Whole-genome sequencing was performed in all samples, confirming the same specimen, which belonged to the B.1.177 lineage.

The first dose of the COVID-19 mRNA vaccine was administered between the fifth and sixth chemotherapy cycles, which were resumed after the first negative SARS-CoV-2 PCR.

## Discussion

This case stands out for the long viral persistence of seven months and the various episodes of clinical relapse of severe COVID-19 related to the worsening immune status with each cycle of R-CHOP. Viral sequencing distinguished persistent infection from reinfection. The literature suggests that the antibody-mediated ablation of B-cell precursors by rituximab is primarily responsible for this prolonged viral shedding [7,8]. Among published case reports, SARS-CoV-2 viral shedding was documented for a year in a patient with B-cell depletion, and for 119 days in another with mantle cell lymphoma and associated B-cell immunodeficiency, with episodes of clinical relapse after the index hospitalization documented in both [8,9]. This prolonged viral shedding represents a risk both for the patient and for close contacts, particularly in the hospital setting. Viral cultures are unavailable in our region, but the several relapses in the presence of similar viral loads allow us to presume the virus shed was continuously infectious. In immunocompromised hosts, some authors recommend a 20-day isolation after symptom onset, or first positive viral test if asymptomatic, while others defend a test-based strategy [10,11]. The best approach to ending isolation is, therefore, challenging.

Management of severe COVID-19 in cancer patients is overall similar to other patients. Antiviral therapy with remdesivir is associated with faster recovery and reduced risk of mechanical ventilation in severe patients not on ventilatory support [12]. In hospitalized patients with hematologic malignancies, it may additionally be combined with passive immunization, if available [7]. In this subset of patients, the administration of convalescent or vaccinated plasma resulted in faster time to recovery and improved survival, easily understood by their baseline hypogammaglobulinemia from both cancer and treatment [13]. In our case, vaccination was not yet widely distributed at the time, so we opted for the administration of polyclonal immunoglobulins.

For most patients with COVID-19, cancer-specific treatment should be interrupted and typically resumed once transmission-based precautions are discontinued, or earlier if symptoms are mild or improving [7,14]. In our patient, given the long viral persistence and the various hospitalizations for severe COVID-19, and considering the apparent cancer remission, we opted for immunochemotherapy suspension until viral eradication and radiological resolution. With this additional measure, the patient had no further hospitalizations related to COVID-19.

## Conclusions

Immunocompromised hosts with COVID-19 have a higher risk of severe disease and shed viable viruses for prolonged periods of time, especially those with hematologic malignancies on anti-CD20 treatment. This case stands out for the long viral persistence of seven months and the various relapses of severe COVID-19 related to the worsening immune status with each cycle of R-CHOP. It represented a clinical challenge and a risk for both the patient and the community, with important public health and infection control implications.
